# Brain metastases from breast cancer: prognostic significance of HER-2 overexpression, effect of trastuzumab and cause of death

**DOI:** 10.1186/1471-2407-11-395

**Published:** 2011-09-19

**Authors:** Romuald Le Scodan, Ludivine Jouanneau, Christophe Massard, Maya Gutierrez, Youlia Kirova, Pascal Cherel, Julie Gachet, Alain Labib, Emmanuelle Mouret-Fourme

**Affiliations:** 1 Department of Radiation Oncology, Centre Hospitalier Privé Saint Grégoire, Saint Grégoire, France; 2 Departments of Radiation Oncology, Institut Curie-Hôpital René Huguenin, Saint Cloud, France; 3 Medical Statistics, Institut Curie-Hôpital René Huguenin, Saint Cloud, France; 4 Medical Oncology, Institut Curie-Hôpital René Huguenin, Saint Cloud, France; 5 Radiology, Institut Curie-Hôpital René Huguenin, Saint Cloud, France; 6 Department of Medical Oncology, Institut Gustave Roussy, Villejuif, France

**Keywords:** brain metastases, breast cancer, trastuzumab, whole brain radiation therapy

## Abstract

**Background:**

To access the prognostic significance of HER-2 overexpression, the effect of trastuzumab and the cause of death in patients with brain metastases (BM) from breast cancer (BC).

**Methods:**

We analyzed the outcome of 130 patients with BM from BC who received whole-brain radiotherapy (WBRT) (without surgery or radiosurgery) between January 1998 and April 2006. Demographic data, tumor characteristics, and treatments were prospectively recorded. The impact of HER-2 overexpression and trastuzumab-based therapy on overall survival (OS) and the cause of death were evaluated.

**Results:**

The median follow-up for the whole population was 6.25 months (mean: 9.15; range: 0.23-53). The median survival time and 1-year survival rates after BM diagnosis were 7.43 months and 35.8% (95% CI: 28-45.7) respectively. The median survival time for HER-2 negative patients (n = 78), HER-2 positive patients not treated with trastuzumab (n = 20) and HER-2 positive patients treated with trastuzumab (n = 32) were 5.9 months, 5.6 months and 19.53 months, respectively. The 1-year survival rates were 26.1%, 29.2% and 62.6% respectively, (p < 0.004). Among the 18 HER-2 positive patients treated with trastuzumab who died, 11 (61%) apparently succumbed from CNS progression, in the face of stable or responsive non-CNS disease. Trastuzumab-based therapy was associated with a 51% reduction in the risk of death (multiadjusted hazard ratio: 0.49; 95% CI, 0.29-0.83).

**Conclusions:**

In our experience, trastuzumab-based therapy for HER-overexpressing tumors was associated with improved survival in BM BC patients. This subgroup of patients may benefit from innovative approaches, in order to obtain better intra cerebral control.

## Background

About 10% to 30% of patients with metastatic breast cancer develop brain metastases (BM) [1]. Several reports suggest that the risk of developing BM is higher (25% to 40%] in patients receiving trastuzumab-based therapy for HER2-overexpressing metastatic breast cancer [2-9]. Whole-brain radiotherapy (WBRT] is considered the standard treatment for most patients, particularly those with extensive intra-cranial disease, providing symptom relief and prolonging both median and overall survival (1,10-12). Despite the use of WBRT, the prognosis of patients with BM remains poor, with a median survival time of approximately 5 months [1,10-14]. Recent studies have examined the influence of patient characteristics on survival in this setting and have attempted to identify subgroups of patients with substantially different outcomes in order to tailor therapy and to rationalize the design, stratification and interpretation of clinical trials [13-19]. The Radiation Therapy Oncology Group (RTOG] recursive partitioning analysis (RPA) classification based on clinical factors (Karnofsky performance status, age, and control of extracerebral disease) is a major prognostic indicator for patients with brain metastases [13]. Several reports suggest that trastuzumab-treated HER2-positive breast cancer patients with BM fare better than HER2-negative breast cancer patients and patients with HER2-positive tumors who do not receive trastuzumab [20-26]. The prognostic significance of HER-2 overexpression and trastuzumab-based therapy has not been analyzed in the previously published prognostic scores of patients with brain metastases. The aim of this study was to confirm, in a cohort of patients with BM from breast carcinoma, the beneficial effect of trastuzumab in patients with HER2-positive disease, and to analyze the cause of death.

## Methods

### Patients and treatments

Between January 1998 and April 2006, 195 consecutive breast cancer patients with BM were treated at Institut Curie-Hôpital René Huguenin Cancer Center, Saint Cloud, France. The study population consisted of 130 patients who received whole brain radiation therapy (WBRT) (without surgery or radiosurgery) and whose tumoral HER-2 status was known. The characteristics of these 130 patients, their tumors, metastatic sites, and therapy (chemotherapy, endocrine therapy or trastuzumab-based therapy) were prospectively recorded in the hospital's MEDICOD database. Karnofsky performance status (KPS) (< 70 vs ≥ 70), the Radiation Therapy Oncology Group (RTOG) recursive partition analysis (RPA) class (I-II vs III) [13] and the number of BM (single vs multiple) at the time of BM diagnosis were obtained retrospectively from the medical charts. The primary tumor was considered to be HER-2-positive (HER-2+) if it scored 3+ on immunohistochemistry (IHC), or if it scored 2+ on IHC and showed gene amplification by fluorescence in situ hybridization (FISH). Trastuzumab exposure for metastatic disease before and after BM diagnosis was recorded. All the patients had computed tomography (CT) and/or magnetic resonance imaging (MRI) for BM diagnosis. WBRT was delivered with a standardized lateral opposed fields technique that used 6-MV or 10-MV photons, up to a standard dose of 30 Gy in ten daily 3-Gy fractions. The patients were seen every month for 6 months after the end of treatment, and then every 2 months. Our institutional review board approved the acquisition, analysis and reporting of the patients' data.

### Statistical analysis

Patient characteristics were compared by using the chi-square test or Ficher's exact test for categorical variables and by using the T-test or Kruskall Wallis test for the quantitative variables. Overall survival was defined as the time from BM diagnosis to the last visit or death. Survival curves were constructed with the Kaplan Meier method [27] and compared with the log-rank test. Multivariate analysis (Cox regression model) tested the following variables for their impact on overall survival: age at BM diagnosis, KPS (< 70 vs ≥ 70), RTOG RPA class (I-II vs III), presence of extracranial metastases, sites of other extracranial metastases (bone vs lung vs liver vs multiple), number of BM (single vs multiple), interval between primary tumor and BM diagnosis (< 2 years vs > 2 years), tumor HR status, lymphocyte count at BM diagnosis (< 0.7 vs > 0.7 × 10^9^/L), HER-2 overexpression and trastuzumab-based therapy. First, the variables were obtained in univariate analysis. Then, the multivariate model was computed with backward step. Differences with P values < 0.05 were considered statistically significant.

## Results

### Patient characteristics and treatments

The characteristics of the 130 eligible patients are reported in table 1. Briefly, mean age at diagnosis was 52.8 years (median: 52; range: 26-83), and 54 patients (41.5%) were younger than 50 years. The KPS was ≥ 70 in 62.2% of cases. The median time from breast cancer diagnosis to BM diagnosis was 40.6 months (range, 0-265 months). Fifty-two patients (40%) had tumors that overexpressed Her-2, and 32 patients had received trastuzumab-based therapy in the metastatic setting. Patients treated before 2001 were not systematically treated with trastuzumab. Of these 32 pts, 5 pts stopped trastuzumab before the diagnosis of BM because of systemic progression, 5 discontinued trastuzumab at the diagnosis of BM and 22 continued a trastuzumab-based therapy after WBRT.

**Table 1:** patient characteristics at diagnosis of brain metastases and treatments

Patient and tumor characteristics	HER-2 negative patients	HER-2 positive patients not treated with trastuzumab	HER-2 positive patients treated with trastuzumab	Whole population	P value
	N = 78	N = 20	N = 32	N = 130	
	-60%	-15.38%	-24.62%		
Age at BM diagnosis (years)- median (range)					
< 65 years	54(34-83)	53 (33- 75)	47(26-67)	52(26-83)	0.0178
≥ 65 years	59/77 (76.62)	15/20 (75)	31/32 (96.88)	105/129 (81.4)	
	18/77 (23.38)	5/20 (25)	1/32 (3.12)	24/129 (18.6)	
Karnofsky performance status					
< 70	32/76 (42.11)	10/20 (50)	6/31 (19.35)	48/127 (37.8)	0.0418
≥ 70	44/76 (57.89)	10/20 (50)	25/31 (80.65)	79/127 (62.2)	
RTOG RPA class	0	0	0	0	0.0391
I	43/75 (57.33)	10/20 (50)	25/31 (80.65)	78/126 (61.9)	
II	32/75 (42.67)	10/20 (50)	6/31 (19.35)	48/126 (38.1)	
III					
Histology of primary BC					
Ductal	70/76 (92.11)	19/20 (95)	30/32 (93.75)	119/128 (92.97)	0.5172** (ns)
Lobular	2/76 (2.63)	0	2/32 (6.25)	4/128 (3.12)	
Other	4/76 (5.26)	1/20 (5)	0	5/128 (3.91)	
Histologic grade					
SBR I	2/70 (2.86)	1/17 (5.88)	1/31 (3.23)	4/118 (3.39)	0.063** (ns)
SBR II	27/70 (38.57)	1/17 (5.88)	11/31 (35.48)	39/118 (33.05)	
SBRIII	41/70 (58.57)	15/17 (88.24)	19/31 (61.29)	75/118 (63.56)	
Tumor HR status					
Positive	48/76 (63.16)	9/19 (47.37)	17/32 (53.12)	72/127 (56.69)	0.1996 (ns)
Negative	28/76 (36.84)	10/19 (52.63)	15/32 (46.88)	55/127 (43.31)	
Number of BM					
Single	6/78 (7.69)	0	0	6/130 (4.62)	0.1339** (ns)
Multiple	72/78 (92.31)	20/20 (100)	32/32 (100)	124/130 (95.38)	0.1778 **(ns)
Meningitis	17/77 (22.08)	1/20 (5)	4/31 (12.9)	22/128 (17.19)	
Yes	60/77 (77.92)	19/20 (95)	27/31 (87.1)	106/128 (82.81)	
No					
Number of other metastatic sites					
0 (BM alone)	6/78 (7.69)	1/20 (5)	0	7/130 (5.38)	0.2971** (ns)
1	19/78 (24.36)	3/20 (15)	9/32 (28.12)	31/130 (23.85)	
2	20/78 (25.64)	7/20 (35)	14/32 (43.75)	41/130 (31.54)	
> 2	33/78 (42.31)	9/20 (45)	9/32 (28.12)	51/130 (39.23)	
Concomitant and other metastases					
Liver	35/78 (44.87)	12/20 (60)	23/32 (71.88)	70/130 (53.85)	0.0299
Lung	36/78 (46.15)	11/20 (55)	14/32 (43.75)	61/130 (46.92)	0.7147 (ns)
Bone	51/78 (65.38)	10/20 (50)	18/32 (56.25)	79/130 (60.77)	0.3783 (ns)
Previous chemotherapy	55/78 (70.51)	18/20 (90)	32/32 (100)	105/130 (80.77)	0.0002**
Yes	23/78 (29.49)	2/20 (10)	0	25/130 (19.23)	
No					
Previous taxane-based chemotherapy	36/78 (46.15)	11/20 (55)	25/31 (80.65)	72/129 (55.81)	0.0047
Yes	42/78 (53.85)	9/20 (45)	6/31 (19.35)	57/129 (44.19)	
No					
Lactate Deshydrogenase (LDH)	35/61 (57.38)	9/14 (64.29)	13/19 (68.42)	57/94 (60.64)	0.6597 (ns)
≤ 500 UI/L	26/61 (42.62)	5/14 (35.71)	6/19 (31.58)	37/94 (39.36)	
500 UI/L	127-2088	120-3469	158-2199	120-3469	
range					
Lymphocyte count	25/70 (35.71)	9/20 (45)	3/20 (15)	37/110 (33.64)	0.1106 (ns)
≤ 0.7 G/L	45/70 (64.29)	11/20 (55)	17/20 (85)	73/110 (66.36)	
> 0.7 G/L	61-2285	40-1814	63-2050	40-2285	
range					
Vital Status	16/78 (20.51)	2/20 (10)	14/32 (43.75) 18/32 (56.25)	32/130 (24.62)	0.0138**
alive	62/78 (79.49)	18/20 (90)		98/130 (75.38)	
dead					

Abbreviations: BM = brain metastases; BC = breast cancer; SBR = Scarff-Bloom-Richardson grade; HR = hormone receptor; RTOG = Radiation

** Fisher's exact test

Values are numbers (percentage), unless otherwise stated.

At BM diagnosis, patients with HER2-overexpressing breast cancer treated with trastuzumab-based therapy (n = 32), compared with HER-2 negative patients (n = 78) and HER-2 positive patients not treated with trastuzumab-based therapy (n = 20), were younger (median age:47 vs 54 and 53, respectively; p = 0.01), had a better Karnofsky performance status (KPS ≥ 70: 80.6% vs 57.9% vs 50%, respectively; p = 0.04) and were thus less likely to be RTOG RPA class III, were more likely to have liver metastases (71.88% vs 44.87% vs 60%, respectively; p = 0.02) and were more likely to have received chemotherapy, including taxane-based chemotherapy, for metastatic breast cancer, respectively (table 1). The median dose of WBRT was 30 Gy, in ten 3-Gy daily fractions (mean, 28.1 Gy; range, 3-40 Gy), distributed as follows: 112 patients received 30 Gy in 10 fractions; 13 patients received < 30 Gy in 3-Gy daily fractions; and 5 patients received > 30 Gy in 2-Gy daily fractions. All but 13 of the patients completed the full course of WBRT; these 13 patients discontinued treatment because of deteriorating systemic or brain disease. Most patients received corticosteroids before WBRT started. Chemotherapy had been administered to 105 (80.8%) patients before BM diagnosis.

### Patient outcomes

**Figure 1:**
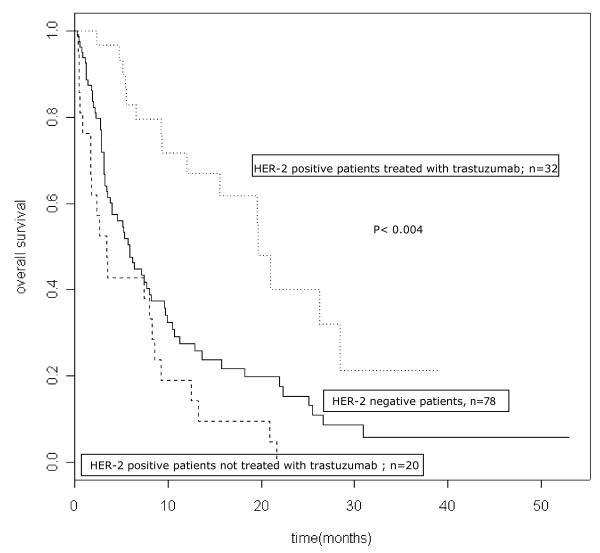
**Survival curves according to HER-2 status and delivery of trastuzumab-based therapy**.

The median follow-up for the whole population was 6.25 months (mean: 9.15; range: 0.23-53). Ninety-eight patients died (75.4%). The median survival time and the 6-month and 1-year survival rates after BM diagnosis were 7.43 months (95% CI: 5.52-9.73), 54.9% (95% CI:46.8-64.3) and 35.8% (95% CI: 28-45.7) respectively. The median survival time among the 79 patients in RPA Class I-II at BM diagnosis was 9.63 months [95% CI: 7.69; 15.72], compared with 3.52 months [95% CI: 2.86; 7.36] among the 48 patients in Class III (p = 0.001]. The median survival time for HER-2 negative patients (n = 78), HER-2 positive patients not treated with trastuzumab (n = 20) and HER-2 positive patients treated with trastuzumab (n = 32) were 5.9 months, 5.6 months and 19.53 months respectively. The 1-year survival rates were 26.1% (95% CI: 16.8-40.7), 29.2% (95% CI:17-50.2) and and 62.6% (95% CI: 47.2-83) respectively, (p < 0.004) (Table 2, Figure 1). The median survival time for the 10 HER-2 positive patients who stopped trastuzumab before or after the diagnosis of BM and the 22 patients who continued a trastuzumab-based therapy after WBRT were 9.2 months and 20.9 months respectively (p > 0.1). The 1-year survival rates were 43.6 (95% CI: 21.8-87.4) and 87.1 (95% CI: 71.8-100), respectively (p = 0.13).

**Table 2:** Rates of overall survival at 6 months and 1 year according to the RTOG RPA class, the HER-2 status and the delivery of trastuzumab

	n	Median Survival (months)	6 months [CI95%]	1 year [CI95%]	P value
Whole population	130	7.43 (5.52 -9.73)	54.9 [46.8; 64.3]	35.8 [28; 45.7]	
RTOG RPA				44.6 [34.3; 58.1]	0.001
Class I-II	78	9.63 (7.69-15.72)	65.9 [56; 77.5]	23.9 [14.2; 40.4]	
Class III	48	3.52 (2.86-7.36)	37.7 [26; 54.7]		
					0.004
HER-2 negative patients	78	5.88 [3.75; 9.63]	48.7 [37.7; 63]	26.1 [16.8; 40.7]	
HER-2 positive patients not treated with trastuzumab	20	5.65 [2.60; 12.49]	45.5 [31.3; 66.1]	29.2 [17-50.2]	
HER-2 positive patients treated with trastuzumab	32	19.53 [9.27; NA]	77.1 [63.5; 93.6]	62.6 [47.2; 83]	
Trastuzumab	10	9.17 (5.52-NA)	63.6 [40.7; 99.5]	43.6 [21.8; 87.4]	0.113
Stopped	22	20.91 (19.53-NA)	94.4 [84.4; 100]	88.5 [74.8; 100]	
Continued					

In univariate analysis, KPS < 70 or RTOG RPA Class III, trastuzumab-based therapy for HER-2-overexpressing tumors, a triple-negative phenotype, Scarff-Bloom-Richardson grade, the serum LDH level and the lymphocyte count at BM diagnosis were predictive of overall survival (Table 3). The following characteristics had no prognostic value: number of BM, sites of other systemic metastases, interval between primary tumor and BM diagnosis, total dose of WBRT, and histology of the primary breast tumor. Trastuzumab-based therapy for HER-2-overexpressing tumors and RTOG RPA Class III or KPS < 70 emerged as independent prognostic factors in multivariate analysis (table 4). Trastuzumab -based therapy was associated with a 51% reduction in the risk of death (multiadjusted hazard ratio [HR] 0.49; 95% CI, 0.29 to 0.83].

**Table 3:** Predictors of overall survival in patients with brain metastases from breast cancer (univariate analyses)

Covariate	Comparison	Hazard Ratio [CI 95%]	P N = 130
Age at BM diagnosis	≥ 50 Vs. < 50	1.21 [0.80; 1.81]	0.329

KPS	≥ 70 vs. < 70	0.51 [0.34; 0.77]	0.0013

RTOG RPA class	III vs. I-II	1.98 [1.32; 2.98]	0.0013

Time interval (y) between primary tumor and BM diagnosis	≥ 2 vs. < 2	3.76 [0.52; 27.18]	0.968

Brain metastases	Presence of systemic metastases vs. Alone	1.52 [0.62; 3.74]	0.366

Visceral metastases	Yes vs No	1.34 [0.84; 2.11]	0.2159

Bone metastases	Yes vs No	1.07 [0.71; 1.60]	0.745

No. of BM	Multiple vs. single	1.12 [0.41; 3.06]	0.822

Nb of other metastatic sites	Multiple vs. single	1.516 [0.6148; 3.74]	0.3627

Histology	lobular and other vs. ductal	0.77 [0.34; 1.77]	0.545

Tumor HR status	Negative vs. positive	1.37 [0.91; 2.06]	0.127

HER-2 overexpression	Negative vs. positive	1.48 [0.98; 2.24]	0.0606

Trastuzumab-based therapy for HER2 overexpressing tumor	Yes vs No	0.45 [0.27; 0.75]	0.001631

HR- & HER2-	Yes vs No	2.17 [1.38; 3.43]	0.0006

SBR grade	3 vs. 1-2	1.59 [1.00; 2.52]	0.0484

LDH (U/L)	> 500 vs. ≤ 500	2.01 [1.27; 3.16]	0.0026

Lymphocyte count	> 700 vs. ≤ 700	0.58 [0.37; 0.89]	0.0138

Total radiation dose (Gy)	> 30 vs. ≤ 30	1.54 [0.49; 4.91]	0.463

Abbreviations: KPS: Karnofsky performance status; HR: hormonal receptor; SBR = Scarff-Bloom-Richardson grade; HR = hormone receptor; RTOG = Radiation

Therapy Oncology Group; RPA = recursive partitioning analysis;

**Table 4:** Multivariate Analysis of Overall Survival, Cox Model (n = 130)

Covariate	Comparison	Hazard Ratio for death [CI 95%]	P N = 127
HER-2 positive patients treated with trastuzumab	Yes vs No	0.49 [0.29;0.83]	0.00810
KPS	≥ 70 vs. < 70	0.56 [0.37; 0.85]	0.00578

Abbreviations: KPS: Karnofsky performance status

### Response to WBRT and cause of death

Among the 32 HER-2 positive patients treated with trastuzumab, only 23 pts had at least one follow-up brain CT scan or MRI by which the radiographic response could be assessed. Ten patients had CNS progression and 13 patients had stable intracerebral disease at last follow-up. The mean PFS in the brain, calculated from the date of WBRT to the date of brain progression, was 7.4 months (range: 1-18). Among the 18 HER-2 positive patients treated with trastuzumab who died, 11 (61%) apparently succumbed from CNS progression, in the face of stable or responsive non-CNS disease. Only 6 HER2 positive BC patients treated without trastuzumab and 30 HER-2 negative patients had at least one follox-up brain CT scan.

## Discussion

Because several subgroups of metastatic breast cancer patients are at a high risk of developing BM [5,9] and because systemic therapy, particularly trastuzumab, has limited efficacy for preventing or controlling intracranial metastases, BM are becoming a major issue in this setting, being associated with poor survival and quality of life [28,29]. Risk factors for the development of CNS metastases from breast cancer include patient characteristics, such as young age and African-American ethnicity, and biological features of the tumor, including ER-negativity, HER2-positivity, high tumor grade, and BRCA1 phenotype [1,7,30-32]. Breast cancer patients with brain metastases form a heterogeneous population with respect to their prognosis [14,20-26]. Identification of patient subgroups with substantially different outcomes is therefore necessary to tailor therapy and to help with the design, stratification and interpretation of future clinical trials. The RTOG RPA classification is currently recognized as a major prognostic indicator in patients with brain metastases. It comprises three prognostic classes based on Karnofsky performance status, age, and control of extracerebral disease [13]. In this classification, the best survival (RPA Class I), with a median of 7.1 months, was observed in patients younger than 65 years of age with a KPS of at least 70, a controlled primary tumor, and metastases restricted to the brain. The worst survival (RPA Class III), with a median of 2.3 months, was seen in patients with a KPS below 70. The remaining patients (RPA Class II) had a median survival of 4.2 months. Several other prognostic scores for patients with brain metastases from different primaries [14-19] were subsequently developed, but none of them include molecular features or breast cancer-specific parameters such as tumor HER-2 overexpression, nor specific treatments.

Several groups have published retrospective studies describing improved survival from time of BM diagnosis in BM patients with HER2-positive BC treated with trastuzumab, compared with HER2-negative breast cancers [20-26]. In a retrospective study including 56 patients with HER2-positive BC who developed BM, Nam and colleagues reported a median OS of 13 months in 21 patients who received trastuzumab after diagnosis of mCNS disease compared with 4 months in those (n = 35) who did not receive trastuzumab after diagnosis and 3 months in 70 BM patients with HER2-negative tumors (p = 0.0011) [26]. Bartsch and colleagues also analyzed the effect of the continuation of trastuzumab after diagnosis of BM for 17 patients, in comparison with a cohort of 36 pts with HER2 overexpressing tumors not treated with trastuzumab after WBRT [20]. In this report, KPS and trastuzumab were associated with better overall survival, with a trend towards longer time to in-brain progression. Our results confirm the fact that trastuzumab-treated HER2-positive breast cancer patients with BM fare better than HER2-negative breast cancer patients and patients with HER2-positive tumors who do not receive trastuzumab [20-26]. In agreement with three previous reports, this survival advantage for patients with brain metastases from tumors that overexpress HER2 does not seem to be due to an intrinsic biologic advantage of HER2 overexpression, as patients with HER2-overexpressing tumors who did not receive trastuzumab had survival similar to that of patients with tumors that did not overexpress HER2 [23,26,33]. In our experience, about 60% of HER-2 positive patients treated with trastuzumab who died apparently succumbed from CNS progression. Similarly, in a retrospective series of 122 patients treated with trastuzumab between 1998 and 2000 at Dana-Farber/Partners Cancer Care, about 50% of the 21 patients with brain metastases who died apparently succumbed from CNS progression, despite stable or responsive non-CNS disease [34]. These results suggest that HER2 targeting may improve brain metastasis outcomes through durable control of systemic extracranial disease in HER2-positive breast cancer patients [35]. However, one should keep in mind that the determination of the cause of death is difficult in this context. As systemic therapies improve, there is concern that the incidence of symptomatic brain metastases will increase, and that control of CNS disease will become a more vital component of overall disease control and quality of life. Althought the clinical activity of trastuzumab on brain metastases remains debated, trastuzumab may cross the blood-brain barrier when its permeability is increased, as it occurs during WBRT [36]. Preclinical results also found that trastuzumab may act synergistically with radiation in a HER2 level-dependent manner [37], encouraging further assessment in combination with WBRT. Chargari and colleagues reported preliminary results of WBRT with concurrent trastuzumab for treatment of BM in 31 BC patients [38]. After WBRT, radiologic responses were observed in 23 patients (74.2%), including 6 (19.4%) with a complete radiologic response and 17 (54.8%) with a partial radiologic response. No Grade 2 or greater acute toxicity was observed. The median survival time from the start of WBRT was 18 months [range, 2-65] and the median interval to brain progression was 10.5 months [range, 2-27]. By dual inhibition of HER1 and HER2, lapatinib also demonstrated a modest efficacy in HER2-positive breast cancer patients with brain metastases [39]. However, Lin et al. found that up to 20% patients with CNS progression after cranial radiation receiving lapatinib and capecitabine experience an objective response [40]. Lapatinib is currently under investigation in combination with WBRT in an ongoing, phase I trial [41]. A phase II study is also currently assessing the efficacy and safety of concurrent WBRT and capecitabine followed by combination capecitabine and sunitinib for treatment of brain metastases from breast cancer [42]. Concurrent chemoradiation is also currently being investigated with the aim of improving the control rate of both cerebral and systemic disease [39,43]. Regarding WBRT fractionation, several trials have examined the influence of the dose, timing and fractionation of WBRT, without identifying a clearly superior schedule [11]. The most widely used WBRT regimen delivers 30 Gy in ten 3-Gy fractions, but this may be inadequate for long-term tumor control. Li et al. recently examined the outcomes of 208 patients who received WBRT as 10 daily 3-Gy fractions in the control arm of the PCI-P120-9801 phase III trial of motexafin gadolinium [44]. Neurocognitive function (NCF) and survival were compared in 135 patients assessable at 2 months with tumor shrinkage below (poor responders) and above (good responders) the population median (45%) on MRI. Good responders survived significantly longer than poor responders (median 300 ± 26 vs 240 ± 19 days; p = .03), and had a longer median time before NCF deterioration, supporting the use of techniques to maximize intracranial control, particularly for patients with HER-2-overexpressing tumors treated with trastuzumab. In our experience, BM patients with HER-2 overexpressing tumors treated with trastuzumab can expect a median overall survival time of about 20 months and a 1-year survival rate of about 60%. This subgroup of patients may therefore have a higher risk of experiencing late radiation-related toxicity such as neurocognitive dysfunction, and might benefit from longer-course WBRT with lower doses per fraction [45]. Further studies of more aggressive local therapy with different dose and fractionation schedules, in a manner that could minimize late effects, could probably be more efficient for this subgroup of patients supposed to have longest median survival.

The combination of a tumor type that has a high potential for CNS spread and a treatment that does not penetrate the CNS but is very effective outside of the CNS creates the opportunity for CNS disease to become a major clinical problem. As an example, the introduction of trastuzumab has altered the natural history of patients with HER2-positive breast cancer, and unmasked CNS metastases as a potential sanctuary site. It is anticipated that this HER2 paradigm is applicable across tumor types, as patients live longer with advanced cancer. In the next decade, there will be a greater need to conduct carefully designed trials of both cytotoxic chemotherapeutic agents and targeted agents, either alone, or in combination, with specific CNS end points.

## Conclusions

In conclusion, BM patients with breast cancer are an heterogenous group of patients. BM patients with HER-2 overexpressing tumors treated by trastuzumab appears to be a clearly distinct subroup of patients who can expect a median survival time of about 20 months and a1-year survival rate of 60%. This information may be useful to tailor the therapy for subgroups of patients, to define homogeneous cohorts for prospective randomized trials, and to identify more precisely patients with relative good prognosis who could be treated with innovative approaches, in order to obtain better intra cerebral control, in a manner that could minimize late effects.

## Competing interests

The authors declare that they have no competing interests.

## Conflict of interest statement

The authors indicate no potential conflict of interest.

## Authors' contributions

RLS and CM designed coordinated the study and drafted the wrote the manuscript. LJ and EMF performed the statistical analysis. MG, YK PC, JG and AL participated in its design and coordination and helped draft the manuscript. All authors read and approved the final manuscript.

## Pre-publication history

The pre-publication history for this paper can be accessed here:

http://www.biomedcentral.com/1471-2407/11/395/prepub
